# Predictors of performance on the pediatric board certification examination

**DOI:** 10.1186/s12909-021-02515-z

**Published:** 2021-02-22

**Authors:** Osamu Nomura, Hirotaka Onishi, Yoon Soo Park, Nobuaki Michihata, Tohru Kobayashi, Kazunari Kaneko, Tetsushi Yoshikawa, Akira Ishiguro, Japan Pediatric Society Steering Committee of Board Examination

**Affiliations:** 1 Department of Emergency and Disaster Medicine, Hirosaki University, 5 Zaifu, Hirosaki, Aomori, 036-8216 Japan; 2 Center for Postgraduate Education and Training, National Center for Child Health and Development, 2-10-1 Okura, Setagaya-ku, Tokyo, Japan; 3 International Research Center for Medical Education, The University of Tokyo, 7-3-1 Hongo, Bunkyo-ku, Tokyo, Japan; 4 Harvard Medical School, Harvard University, 25 Shattuck St, Boston, MA USA; 5 Massachusetts General Hospital, 55 Fruit Street Bartlett (BAR-2R-202), Boston, MA USA; 6 Department of Health Services Research, The University of Tokyo, 7-3-1 Hongo, Bunkyo-ku, Tokyo, Japan; 7 Department of Management and Strategy, Clinical Research Center, National Center for Child Health and Development, 2-10-1 Okura, Setagaya-ku, Tokyo, Japan; 8 Department of Pediatrics, Kansai Medical University, 2-5-1 Shin-machi, Hirakata, Osaka, Japan; 9 Department of Pediatrics, Fujita Health University, 1-98 Dengakugakubo, Kutsukake-cho, Toyoake, Aichi Japan; 10 Japan Pediatric Society Steering Committee of Board Examination, 1-1-5, Koraku, Bunkyo-ku, Tokyo, Japan

**Keywords:** Pediatrics, Predictors, Board certification, Performance, Resident

## Abstract

**Background:**

Examining the predictors of summative assessment performance is important for improving educational programs and structuring appropriate learning environments for trainees. However, predictors of certification examination performance in pediatric postgraduate education have not been comprehensively investigated in Japan.

**Methods:**

The Pediatric Board Examination database in Japan, which includes 1578 postgraduate trainees from 2015 to 2016, was analyzed. The examinations included multiple-choice questions (MCQs), case summary reports, and an interview, and the predictors for each of these components were investigated by multiple regression analysis.

**Results:**

The number of examination attempts and the training duration were significant negative predictors of the scores for the MCQ, case summary, and interview. Employment at a community hospital or private university hospital were negative predictors of the MCQ and case summary score, respectively. Female sex and the number of academic presentations positively predicted the case summary and interview scores. The number of research publications was a positive predictor of the MCQ score, and employment at a community hospital was a positive predictor of the case summary score.

**Conclusion:**

This study found that delayed and repeated examination taking were negative predictors, while the scholarly activity of trainees was a positive predictor, of pediatric board certification examination performance.

**Supplementary Information:**

The online version contains supplementary material available at 10.1186/s12909-021-02515-z.

## Background

Pediatrician competency is crucial for assuring an acceptable quality of pediatric care and improving patient outcomes. Competency-based medical education (CBME), defined as “an outcomes-based approach to the design, implementation, assessment, and evaluation of medical education programs, using an organizing framework of competencies” is a core concept in medical education, and its framework reflects the social accountability regarding patient needs [1]. Evaluating the competency of medical trainees is therefore fundamental to ensuring each trainee’s professional achievement and providing effective feedback to trainees. In postgraduate medical training, board certification examinations serve as summative assessments of residents’ competency which is expected to achieve when completing their training. Examining the predictors of academic performance in pediatric board examinations is also a meaningful way of improving educational programs and constructing appropriate learning environments for residents.

Wakeford et al. investigated predictors of success in postgraduate medical examinations among United Kingdom (UK) trainees, and identified ethnicity as a potential predictor [2]. Scrimgeour et al. also suggested sex, ethnicity, and age as predictors of success in postgraduate surgical examinations in the UK [3]. Furthermore, Australian studies have found that Grade Point Average at the completion of undergraduate studies predicted the workplace performance of junior doctors [4, 5]. While similar studies have been conducted using board certification examinations in other specialties such as general surgery, anesthesiology, and emergency medicine in the United States (US), studies examining these predictors in pediatrics remain limited [6–9]. Performance on in-training examinations during pediatric residency is known to offer a significant predictor of performance in board examinations [10], but investigations into the relationship between trainee-related explanatory variables such as characteristics of residency programs, scholarly activities of residents, and test performance can provide useful contextual indicators to improving learning and residency curricula.

By identifying the predictors of academic performance in pediatric trainee physicians, the faculty of residency programs can implement and promote a support system to help trainees prepare for their board certification examinations. The aim of this study was, therefore, to identify the predictors of postgraduate pediatric trainee performance on board certification examinations.

## Methods

### Context

In Japan, the medical academic society in each specialty has been responsible for the board certification of trainees in accordance with the rules of the Japanese Medical Specialty Board [11]. In pediatrics, the Japan Pediatric Society (JPS) manages the board certification examinations, and trainees who choose pediatrics as a specialty enroll in a pediatric residency program approved by the JPS, which is supervised by a program director whose responsibility is to approve the trainees’ application for the board examination based on various prerequisites, including completion of the residency logbook and a case summary report.

### Assessment categories

The JPS board examination is designed to evaluate trainees’ performance based on the CBME concept of the JPS, which includes three components: (1) multiple-choice questions (MCQs), (2) case summary report, and (3) an interview regarding the submitted case summaries [12]. The written portion of the examination aims to evaluate the examinees’ knowledge of general pediatrics and clinical reasoning skills in pediatric medicine. Examinees are administered 120 MCQs within 3 h in the first session of the board examination.

In case summary assessment, which measures integrative clinical understanding of patient care, child health, and pediatric advocacy, including injury prevention activities, each candidate is required to submit 30 case summaries in designated disease categories, such as infectious, hematological, and endocrine diseases. Examinees submit summaries 3 months prior to the examination date and members of the Steering Committee of Board Examination evaluate these summaries based on the predetermined assessment rubric following a double-blinded review protocol.

Interviews are designed to assess the candidates’ critical thinking skills and professionalism, and two examiners ask the candidates questions based on two case summaries submitted by the candidates themselves. Interviews are held in the second session of the examination after the MCQ test, and the duration of the interview for each examinee is 15 min. Evaluation is performed based on the predetermined assessment rubric.

All three test components are considered as completely separate elements using numeric scores (continuous data). Examinees are required to pass each component in a non-compensatory manner to pass the overall board examination. Cut-off points for each component are determined by the Japan Pediatric Society Steering Committee of Board Examination. This board examination is administered once a year in September, and its annual pass rate is approximately 70 to 80%. Failed examinees are eligible to take the examination in each succeeding year as needed, with no limits imposed on the numbers of times a candidate may take the exam. The pass or fail results were not available for this study due to confidentiality issues.

### Design and participants

**Fig. 1:**
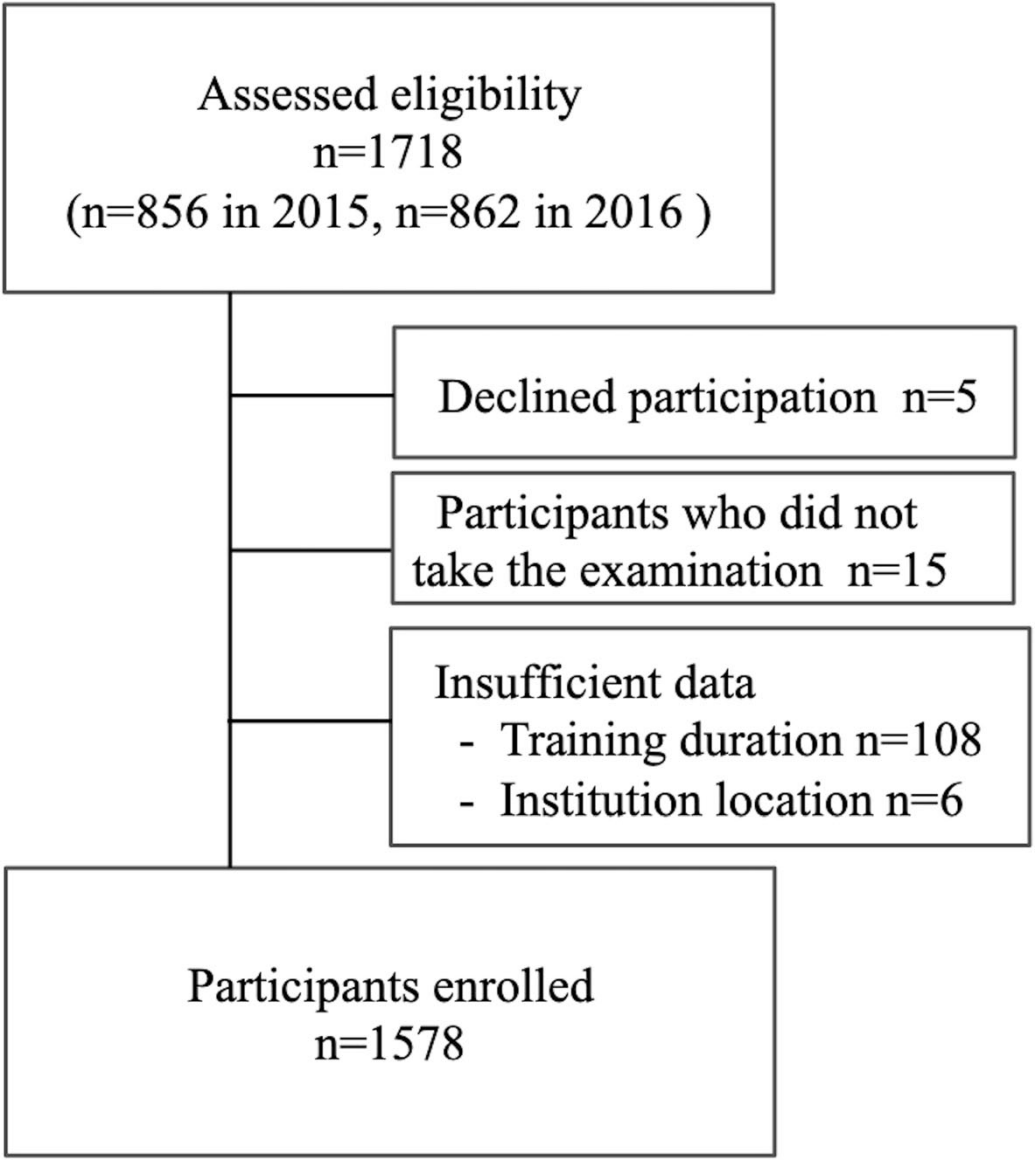
Flow chart of this study

We conducted a secondary analysis of the retrospective examination data by utilizing the JPS board certification database [13]. All pediatricians in Japan who took the pediatric board examination in 2015 and 2016 were recruited in this study, with 856 and 862 pediatricians taking the examination in 2015 and 2016, respectively. Of all 1718 eligible participants, five declined participation, and 129 were excluded due to the omission of data. Consequently, 1578 trainees were enrolled (Fig. 1).

### Data collection

The database information included the trainees’ sex, duration of training, types of training institutions, location of the institutions, number of times the board examination was taken, numbers of research presentations and research publications, and the board examination results. Regarding number of examinations taken, the first attempt was indicated as 1, with pediatricians who failed in their earlier attempt(s) showing 2 or a higher number. These variables were selected for analysis based on evidence from the existing literature. For example, studies from the US and UK have identified the demographic characteristics of the trainees were predictors of the test performance [2–10], and another UK study found variation in exam performance according to the institution to which trainees belonged [14]. In addition, US studies have suggested that number of times the board examination was taken and scholarly activity were associated with the academic performance of the trainees [15, 16].

The training institution categories included national or public university hospitals, private university hospitals, children’s hospitals, and community hospitals. We applied the definition of “urban areas” of the Japanese Medical Specialty Board (i.e., Tokyo, Kanagawa, Aichi, Osaka, and Fukuoka) to determine whether the institutions were located in an urban or non-urban area.

A research presentation was defined as a presentation conducted at an academic conference held by an academic society; thus, conferences within the residents’ institutions, such as case conferences, grand rounds, and morbidity and mortality conferences, were not included. A research publication was defined as an article published in a peer-reviewed journal, including Japanese journals published by Japanese academic societies as well as PubMed-indexed international journals.

### Statistical analysis

We employed descriptive statistics to characterize the participants by sex, number of examination attempts, training duration, types of training institutions, location of the institutions, number of presentations and publications, and the MCQ, case summary, and interview scores. Multiple regression analyses were conducted for the continuous scores of each of the three test components (i.e., separate models were created for each of the MCQ, case summary, and interview tests) as outcomes, and included demographic information from trainees as independent variables, such as sex, number of examination attempts, training duration, type and location of the institution, and scholarly activities. These variables were chosen based on evidence from the literature and statistical evidence such as improvement in model fit and results from the bivariate analysis.

Model fit and possible multicollinearity of predictors were checked using standard diagnostic tools (*F*-statistic, *R*^2^, and variance inflation factor statistics). Statistical analyses were conducted using SPSS version 23.0 (IBM Corporation, 2018, Armonk, NY, USA).

### Ethical aspects

This study was approved by the Ethics Committees of The National Center for Child Health and Development in December 2014 (No. 74) and the JPS in March 2015.

## Results

### Descriptive statistics

The descriptive results are shown in Table 1.

**Table 1:** Demographics of the participants (*n*=1578)

	*M* (*SD*)	% (n)
Female sex		41.0 (647)
Training duration (years)	4.6 (3.0)	
Test attempts (times)	1.5 (1.0)	
Types of training institution		
Public university hospital		34.5 (544)
Private University hospital		31.5 (497)
Children’s hospital		9.1 (144)
Community hospital		24.9 (393)
Residency in urban area		47.6 (751)
Number of academic presentations	4.5 (3.2)	
Number of research publications	0.3 (0.7)	
MCQ score	71.0 (9.6)	
Case summaries score	86.5 (6.1)	
Interview score	88.6 (12.0)	

### Multiple regression analysis

We conducted simultaneous multiple regression analyses to explore the predictors of test performance for each of the three examination components: MCQ, case summaries, and the interview.

#### MCQ model (Table 2)

A multiple regression model for the MCQ score indicated that predictors for sex, training duration, number of examination attempts, type and location of the institution, and research experience explained 14% of the variance in the MCQ score (*F* (9, 1568) = 28.56, *p* < .001). The number of research publications was found to be a significant positive predictor of the MCQ score (*β* = .075, 95%CI: .33 to 1.73). On the other hand, the number of examination attempts (*β* = −.229, 95%CI: − 2.59 to − 1.66), training duration (*β* = −.205, 95%CI: −.81 to −.49) and employment at a community hospital (*β* = −.75, 95%CI: − 2.98 to −.38) were negative predictors of performance.

**Table 2:** A simultaneous multiple regression for variables predicting MCQ score

Variable	*B*	*β*	95% CI
Constant	77.196		75.76 to 78.63
Female sex	.44	.002	−.86 to .95
Training duration (years)	−.651	−.205	−.81 to −.49
Test attempts (times)	−2.124	−.229	−.2.59 to −1.66
Types of training institution			
Public university hospital (Reference)	–	–	–
Private university hospital	−.67	−.032	−1.79 to .45
Children’s hospital	.751	.022	−.93 to .2.43
Community hospital	−1.684	−.75	−2.98 to. -.38
Residency in urban area	−.667	−.035	−1.66 to .32
Number of academic presentations	.142	.047	−.010 to .294
Number of research publications	1.032	.075	.33 to 1.73

*Note*. *R*^2^= .141 (*p* < .05)

#### Case summary report model (Table 3)

A multiple regression model for the case summary report score showed that the variables of sex, training duration, number of examination attempts, type and location of the institution, and research experience explained 10% of the variance in the case summary score (*F* (9, 1568) = 19.39, *p* < .001). Female trainees (*β* = .06, 95%CI: .19 to 1.36), employment at a community hospital (*β* = .06, 95%CI: .01 to 1.70), and the number of academic presentations (*β* = .06, 95%CI: .02 to .22) were significant positive predictors of their case summary score. However, training duration (*β* = −.21, 95%CI: −.53 to −.32), the number of examination attempts (*β* = −.14, 95%CI: − 1.11 to −.50), and employment at a private university hospital (*β* = −.60, 95%CI: − 1.51 to −.06) were negative predictors.

**Table 3:** A simultaneous multiple regression for variables predicting case summary scores

Variable	*B*	*β*	95% CI
Constant	89.66		88.74 to 90.59
Female sex	.77	.06	.19 to 1.36
Training duration (years)	−4.2	−.21	−.53 to −.32
Test attempts (times)	−.80	−.14	−1.11 to −.50
Types of training institution			
Public University hospital (Reference)	–	–	–
Private University hospital	−.78	−.60	−1.51 to −.06
Children’s hospital	−.29	−.01	−1.38 to .80
Community hospital	.085	.06	.01 to 1.70
Residency in urban area	−.12	−.01	−.76 to .52
Number of academic presentations	.12	.06	.02 to .22
Number of research publications	.16	.02	−.30 to .61

*Note*. *R*^2^=. 10 (*p* < .05)

#### Interview Model (Table 4)

A multiple regression model for the interview score indicated that the variables of sex, training duration, examination attempts, types and location of institutions, and research experiences explained 5% of the variance in the interview score, *F* (9, 1568) = 9.14, *p* < .001.

**Table 4:** A simultaneous multiple regression for variables predicting interview scores

Variable	*B*	*β*	95% CI
Constant	92.17		90.30 to 94.04
Female sex	1.60	.07	.42 to 2.79
Training duration (years)	−.25	−.06	−.46 to −.04
Test attempts (times)	−1.75	−.15	−2.36 to − 1.14
Types of training institution			
Public university hospital (Reference)	–	–	–
Private university hospital	.05	.002	−1.40 to 1.51
Children’s hospital	1.41	.03	−.78 to 3.60
Community hospital	−1.07	−.04	−2.76 to .63
Residency in urban area	.32	.01	−.97 to 1.61
Number of academic presentations	.29	.08	.09 to .48
Number of research publications	−.25	−.01	−1.16 to .67

*Note*. *R*^2^=.05 (*p* < .05)

Female sex (*β* = .07, 95%CI: .42 to 2.79) and the number of academic presentations (*β* = .08, 95%CI: .09 to .48) were significant positive predictors of the interview score. On the other hand, training duration (*β* = −.06, 95%CI: −.46 to −.04) and the number of examination attempts (*β* = −.15, 95%CI: − 2.36 to − 1.14) were negative predictors.

### Multicollinearity analysis (Additional file 1)

The correlation between variables and the variance inflation factor (VIF) were determined by multicollinearity analysis. No strong correlation (i.e., > 0.9) was found between the variables and VIF exceeding the recommended cut-off (> 4), indicating that the assumptions for multicollinearity were not violated.

## Discussion

This nationwide study in Japan investigated predictors contributing to performance of pediatric postgraduate trainees on the board certification examination. We found that a longer training duration and previous experiences of failure on the examination were independent negative predictors. We also showed that scholarly activity, such as research presentations and publications represented a positive predictor.

Our study further suggested that trainees who needed a longer time to complete their training showed poor performance. Similar results were obtained for the board examination for pediatrics, emergency medicine, and surgery in the United States and for surgery in the UK [6, 17–19]. These studies revealed that residents who delayed taking the qualifying examination were at high risk of failing to achieve board certification. Three possible explanations of these observations have been adduced: the inability to acquire the program director’s approval to apply for an examination due to insufficient competency of the trainees, natural deterioration of the trainees’ knowledge over time, and other determinants affecting training completion, such as personal or family health issues, anxiety about test performance, and procrastination. Among these potential causes of prolonged training duration, the first explanation might be the most applicable to our setting. Candidates for the pediatric board certification examination are required first to complete 30 case summaries then receive the attending physicians’ feedback [20]. The program director finally approves the trainees’ application based on the revised case summaries. Thus, trainees’ exam-taking may be delayed if they have difficulty completing the summaries and obtaining the approval of the program director. The natural deterioration in the candidates’ knowledge can also lead to a similar result. In Japan, it had not been mandatory for postgraduate trainees to take the board certification examination after their residency until the new board certification system was established across all medical disciplines in 2018 [11]. Therefore, a certain percentage of trainees did not take their board certification examination immediately after completing their training; thus, it might have been difficult for pediatricians who postponed taking the examination to maintain and update their knowledge in general pediatrics.

We also showed that candidates who had taken the examination multiple times performed poorly on their pediatric board certificating examination. Previous Japanese studies indicated that medical students who experienced at least one failure on the national medical license examination tended to fail following examinations multiple times [21]. This finding was also observed for the American Board of Physical Medicine and Rehabilitation certification examinations [15], suggesting that repeated examination-taking is a negative predictor of test performance. In addition, a UK study showed that fewer attempts at the mandatory Membership of the Royal College of Surgeons examination predicted success at the Fellowship of the Royal College of Surgeons (FRCS) examination [19]. The issues of trainees who retake examinations multiple times have been debated in the literature, but a report from the Membership of the Royal Colleges of Physicians (MRCP) exam in the UK found that candidates who took the exams multiple times exhibited improved test performance across multiple attempts [22]. Thus, these results should not be used to limit the number of attempts, but rather to identify those trainees who need special assistance to pass the exam.

While the two negative predictors discussed (delayed and repeated examination-taking) might be correlated, the multicollinearity assumption was not violated, indicating that these two predictors were independent. In terms of the implications of this finding, it is very important for the faculty of pediatric residencies to provide special support to trainees who are extending their training duration or have failed previous examinations. In doing so, each residency program can utilize educational resources effectively and ensure the ability of graduating trainees to competently provide high-quality care to patients.

We also found that scholarly activity, either research presentations or publications, positively predicted performance on the examination. Studies suggests that the experience of publishing studies did not disturb trainees’ academic activities during their residency but instead predicted better performance on the certification examination [23, 24]. Another study reported that publication experience among internal medicine residents significantly correlated with their clinical performance test score [16]. Thus, more experience in research can provide excellent benchmarks for identifying academic leaders in the trainee community, such as chief residents who can help construct appropriate learning environments for other trainees to prepare for the examinations [8, 25]. This can provide opportunities to develop educational systems in each residency program or in board systems that train and graduate competent pediatricians to deliver excellent care to society. One systematic review suggested that educational interventions, such as structured reading programs and a “boot camp” curriculum, were effective for improving the scores in in-training examinations in surgery [26]. Thus, the faculty may be able to provide this peer learning opportunity for trainees requiring special support by collaborating with potential leaders with experience of scholarly activity [27].

Our results show that model fits for multiple regression analyses were not particularly high, explaining 5–14% of variance. However, these models were all statistically significant (*p* < .001), and the current results are in line with those of previous studies. Our findings can thus be considered reliable. Other potential variables may be clinical workplace performance in postgraduate training, such as the Mini-Clinical Evaluation Exercise, Direct Observation of Procedural Skills, and Multiple Source Feedback, as discussed in previous reports [28, 29]. While these variables were not included in the current analysis, the JPS has recently implemented such workplace-based assessment elements to the board training system. These assessment results will thus be able to be incorporated into future analyses of predictors.

There are some limitations to this study. First, this was a nationwide study conducted in Japan, and several features, such as the national board system, are peculiar to our country. While the generalizability of the findings to other circumstances is unclear, our results were consistent with those of previous international studies, indicating possible applicability to other contexts. In addition, although we succeeded in identifying the predictors of examination performance, the pass or fail results were not available due to confidentiality issues, thus possibly limiting the consequential validity of the board certificating examination system. This limitation is expected to be addressed by the renewal of the national board certification system, which aims to ensure the quality and social accountability of each residency program to the Japanese medical community in general. Finally, our analysis did not include pre-existing variables of trainees (such as academic performance in undergraduate medical education) that have been examined in previous studies from other countries. This was because longitudinal cohort databases of medical trainees in Japan are lacking. However, the continuum concept of physician competency has recently been implemented, and workplace-based assessments have been stored in an e-portfolio-type system in Japanese medical education. A longitudinal cohort database may thus be able to be created in the near future.

## Conclusions

This nationwide study in Japan showed that delayed and repeated examination-taking were independent negative predictors of performance on the pediatric board certification examination. These results should enable medical educators to implement educational programs to improve the academic activities of trainees.

## Supplementary Information

**Additional file 1: Appendix A.** Pearson correlation analysis for variables. **Appendix B.** Multicoliniality diagnostics.

## Data Availability

Data are available from the authors upon reasonable request and with permission of the Japan Pediatric Society.

## Abbreviations

CBME: Competency-based medical education
MCQ: Multiple-choice question
CI: Confidence interval
VIF: Variance inflation factor
