# Supplementary material for: Predictors of performance on the pediatric board certification examination
Source: BMC Med Educ. 2021 Feb 22;21:122. doi: 10.1186/s12909-021-02515-z (PMC7898761; doi:10.1186/s12909-021-02515-z)
Supplement: Supplementary file 1 — Additional file 1: Appendix A. Pearson correlation analysis for variables. Appendix B. Multicoliniality diagnostics. [file 12909_2021_2515_MOESM1_ESM.docx]

Appendix A.

*Pearson correlation analysis for variables*

| Variables | 1 | 2 | 3 | 4 | 5 | 6 | 7 | | 8 | | 9 | | 10 | | 11 | | 12 | |  |
| --- | --- | --- | --- | --- | --- | --- | --- | --- | --- | --- | --- | --- | --- | --- | --- | --- | --- | --- | --- |
| 1. Female sex |  |  |  |  |  |  | |  | |  | |  | |  | |  | |  | |
| 2. Training duration (years) | -.02 |  |  |  |  |  | |  | |  | |  | |  | |  | |  | |
| 3. Test attempts (times) | -.06 | .39 |  |  |  |  | |  | |  | |  | |  | |  | |  | |
| 4. Private university hospital | -.05 | -.06 | -.01 |  |  |  | |  | |  | |  | |  | |  | |  | |
| 5. Children’s hospital | -.02 | -.04 | -.03 | -.22 |  |  | |  | |  | |  | |  | |  | |  | |
| 6. Community hospital | .08 | -.02 | .09 | -.39 | -.18 |  | |  | |  | |  | |  | |  | |  | |
| 7. Residency in urban area | .05 | -.10 | .01 | -.05 | .06 | .39 | |  | |  | |  | |  | |  | |  | |
| 8. Number of academic presentations | -.01 | .05 | .02 | -.08 | -.01 | .00 | | -.04 | |  | |  | |  | |  | |  | |
| 9. Number of research publications | -.51 | .20 | .12 | -.05 | -.03 | .00 | | -.02 | | .39 | |  | |  | |  | |  | |
| 10. MCQ score | .01 | -.27 | -.31 | .00 | .05 | -.10 | | -.05 | | .07 | | .026 | |  | |  | |  | |
| 11. Case summaries score | .08 | -.25 | -.21 | -.08 | .00 | .08 | | .04 | | .06 | | -.02 | | .30 | |  | |  | |
| 12. Interview score | .07 | -.13 | -.19 | .01 | .05 | -.05 | | .01 | | .06 | | -.02 | | .13 | | .18 | |  | |

Appendix B.

*Multicoliniality diagnostics*

| Variables | Tolerance | VIF |
| --- | --- | --- |
| 1. Female sex | .99 | 1.02 |
| 2. Training duration (years) | .81 | 1.24 |
| 3. Test attempts (times) | .83 | 1.21 |
| 4. Private university hospital | .73 | 1.37 |
| 5. Children’s Hospital | .84 | 1.20 |
| 6. Community Hospital | .62 | 1.61 |
| 7. Residency in urban area | .80 | 1.25 |
| 8. Number of academic presentations | .84 | 1.19 |
| 9. Number of research publications | .81 | 1.23 |
